# Does Entrepreneurs’ Military Experience Promote Corporate Environmental Investment? Evidence from Chinese Private Firms

**DOI:** 10.3390/ijerph19042104

**Published:** 2022-02-13

**Authors:** Conghua Hong, Youliang Yan, Xinxin Zhang

**Affiliations:** School of Economics and Business Administration, Chongqing University, Chongqing 400030, China; hongconghua@cqu.edu.cn (C.H.); zhangxinxin@cqu.edu.cn (X.Z.)

**Keywords:** military experience, corporate environmental investment, wartime atmosphere, public environmental concern

## Abstract

Although it is well established that the military experience of top executives has a profound influence on firms’ decisions, empirical evidence on how military experience matters to corporate environmental investment has been scarce. Drawn on imprinting theory and upper echelon theory, this study examines the impact of entrepreneurs’ military experience on corporate environmental investment. Using a nationwide survey of Chinese private firms, we find that entrepreneurs’ military experience significantly promotes corporate environmental investment. Further, the positive effect is more pronounced for firms with entrepreneurs who have experienced a wartime atmosphere and those located in regions with more minor public environmental concerns. Our study extends the literature on the determinants of corporate environmental investment and the economic consequences of individual military experience. Moreover, this also provides practical insights for policymakers on how to implement environmental governance and promote ecological construction.

## 1. Introduction

With the rapid development of modern industry, carbon dioxide and other greenhouse gases are emitted in large quantities, causing global warming and posing a severe threat to the survival of human beings and other organisms. In December 2015, 195 countries approved and adopted the first-ever legally binding global climate agreement at the Paris climate conference (COP21). All major carbon emission countries, including China, EU, India, Japan, and Russia, have expressed strong willingness to fulfill their commitments (Appendix A Footnote 1). Since 2010, China has become the world second-largest economy. However, its rapid economic growth seems to be highly coupled with environmental deterioration [1]. In September 2020, the Chinese leader declared that China will reach peak emissions before 2030 and achieve carbon neutrality by 2060 at the United Nations climate conference. Despite the substantial investment, China’s funding gap remains large to achieve the desired results. The total investment in environmental pollution management of China accounted for only 1.15% of the GDP in 2017 (Appendix A Footnote 2). In comparison, environmental investment in developed western nations, which have crossed the peak of governance in environmental protection, still accounted for about 2.5% of GDP. In addition, the current statistics of environmental investment include plenty of funds for landscaping, industrial restructuring subsidies. Accordingly, the actual funds for environmental management projects and operational services are seriously insufficient [2].

As the primary consumers of resources and the leading producers of environmental pollution (Appendix A Footnote 3), enterprises should take the primary responsibility of pollution prevention [3], for their performances play a determining role in the environmental governance of the entire society. Previous studies have noticed the conflict between economy and environment [4]. They are constantly developing sustainable development research, such as studies on environmental entrepreneurship, focusing on how to balance the relationship between the damage to the natural environment and enterprises’ interests through the application of entrepreneurial process [5]. In the process of realizing their environmental value, enterprises need to allocate funds reasonably on the basis of maintaining sustainable operation. In terms of factors influencing corporate environmental investment, previous studies have involved corresponding research on government policies and regulations [6,7,8], public pressure [9,10], religious belief [11], corporate characteristics [12,13], and managerial behavior [14,15]. However, we still know very little about whether and how entrepreneurs’ military experience affects corporate environmental investment. Veterans have turned to business extensively and have become a unique and essential force in the Chinese economy (Appendix A Footnote 4). Military entrepreneurs actively respond to government goals and possess high enthusiasm for social undertakings [16]. Studies have found that corporates, where there are military executives, are more likely to participate in public welfare undertakings such as philanthropy, poverty reduction, and fraud avoidance [16,17,18]. As for environmental governance, entrepreneurs’ military experience may also drive corporates to be more likely to pay for its cost. However, there is currently a lack of corresponding empirical evidence to support the relationship between them, and our study attempts to fill the gap in this regard.

Large number of studies have shown the significant effects of military experience on corporate tax avoidance [19], financing [20], investment [21], acquisitions [22], and firm performance [23]. For individuals, they are typically experiencing role transitions and are forming their worldviews, values, and beliefs during their military service, when they develop characteristics that reflect prominent features of the environment. Military experience leaves a significant imprint, which would possess a long-term and persistent impact on organizational decisions [20], as it shapes their entrepreneurs’ personalities, values, and behaviors [16,17,19,21]. For instance, Malmendier et al. (2011) found that CEOs with military experience pursue more aggressive policies, including heightened leverage [20]. Its effect, however, on executives’ attitudes towards CSR, especially corporate environmental responsibility, has received little attention. This is very important, since corporates are now operating within a social system in which they have direct or indirect relationships with more and more stakeholders. The economic behaviors of firms will have different social consequences [24,25]. Given that the military emphasizes (and trains) obedience, honor, and dedication, military experience may leave a deep value imprint on executives, which exhibits heterogeneous characteristics in company decision-making and produces other social performance. Therefore, the attitude of the military executives towards corporate environmental undertakings is worthy of discussion.

Using the data from the 2012 Chinese Private Enterprise Survey (CPES), we explore the impact of military experience on corporate environmental investment from the perspective of imprinting theory. Our study contributes to the literature in several ways. First, we advance research on the determinants of corporate environmental investment. While most of the respective literature places focus on institutions or governance mechanisms [6,7,26], we complement those studies that show that the character of entrepreneurs is also an essential determinant of that. Furthermore, we add new evidence to the imprinting theory and upper echelon theory. We find that the experience of servicing in the Chinese military, related to obedience, honor, and dedication behavior, has a significantly positive effect on entrepreneurs’ individual value orientation, thus promoting the fulfillment of corporate environmental responsibility. Finally, by taking wartime atmosphere and public environmental concerns into consideration, we provide novel evidence on a more comprehensive understanding of the dynamic change of military imprint under different external circumstances.

The remainder of this study is organized as follows: Section 2 explains the theoretical background and hypotheses. Section 3 provides details of the data, samples, and models used in this study. Section 4 includes empirical results. Section 5 is the discussion of this paper. Finally, Section 6 summarizes the research results and discusses more contributions.

## 2. Literature Review and Hypothesis Development

### 2.1. Theoretical Foundation

Servicemen and women generally join the army after entering adulthood, that is a period in which people are vulnerable to the environment in their lives [27]. Compared with other occupations, military service is more likely to emphasize some characteristics. Evidence from sociological and psychological studies shows that veteran entrepreneurs may show a more optimistic attitude, firmer will, and a value system of integrity, loyalty and morality. Benmelech et al. (2011) found that CEOs who served in the military can make better decisions when facing pressure and crisis [21]. Other literature believe that military experience can lead to individual overconfidence and strengthen risk-taking willingness [20]. However, the research in this field is still limited, far from the “big unification theory,” and more research is needed to understand better the impact of military experience on the beliefs, value orientations, and decision-making of senior managers.

Entrepreneurs of private corporates are not only business owners, but also top managers of their corporate, which means they are prominent decision-makers of environmental investment [28]. The imprinting theory suggests that individuals develop imprints that match their external environment when they go through a sensitive period. Even if the external environment changes, the imprints developed during these particular periods will continuously influence individual behavior [29]. Early experiences have been found to significantly impact the development of entrepreneurs’ cognition traits [20,21], thereby directly affecting the behaviors of individuals. As an exceptional early experience for entrepreneurs, the military experience could affect the deep cognitive features of entrepreneurs [17] and form a deep imprint [30].

Additionally, the Upper Echelons Theory proposed by Hambrick and Mason (1984) argues that managers are finite rational, and heterogeneous. Heterogeneity in managerial experiences affects their values and cognition, leading to differences in corporate decisions and strategic choices, and further to differences in economic outcomes. Personal experiences can effectively shape executives’ personalities, preferences, beliefs, and the way they reflect on current strategic opportunities and issues, that are critical to decision making [31]. Mounting empirical evidence confirms that executives’ experiences during critical periods, like birth or graduation, influence their decision-making behavior, such as risk appetite [32], and management style [33]. Consequently, Upper Echelons Theory, as well as imprinting theory, lays a theoretical foundation for this study to construct a logical relationship between military experience and corporate environmental investment.

### 2.2. Military Experience and Corporate Environmental Investment

When individuals enter a new social organization, there will be a socialization process, in which they acquire new values, knowledge, and skills [34] and develop ongoing behavioral patterns [35]. The norms and cognitive patterns associated with a particular organization imprint on the individuals, as well as shape their values and beliefs. These effects will remain even after they leave the particular organization [36,37].

The imprint of military experience helps to establish multiple values that drive military entrepreneurs to engage themselves more actively in environmental investments during the operation and growth of their businesses. Discipline, obedience, and loyalty are the virtues they may be taught in the first place. Entrepreneurs who grow up under the disciplinary culture of the army will develop the psychological characteristics of obeying rules [38]. This strict military system is manifested not only in the restraint and regulation of soldiers’ behaviors, but also in the rigid hierarchy in which unconditional obedience is required when orders come from superiors. When Adelheid et al. studied Canadian military right-wing authoritarianism, they argued that the military fosters obedience, which leads to establishing rules [39]. Likewise, in the context of talk about military service, Gibson and Condor considered England and Poland are treated as impersonal constructs, ‘fight[ing] for’ one’s country essentially entails obedience to some external, and essentially unpredictable, political authority [40]. As a result of values education, the organization establishment, ideology, and discipline enforced by the Chinese military reinforced the compliance and obedience of entrepreneurs to the institution in their early years and fostered their loyalty [36]. Meanwhile, on the environmental policy front, since the beginning of the Eleventh Five-Year Plan, China has raised the total pollution control to the height of the national environmental protection strategy [3]. Various industrial policies have been introduced, requiring enterprises to actively participate in environmental governance, encouraging them to introduce green production technology, and increasing investment in environmental governance [41]. Thus, the quality of being loyal and obedient to the government, established during the military service, will make a difference in their interpretation and implementation of these policies.

The second is a strong sense of honor. All soldiers of all countries globally value honor. Soldiers are born out of war, which is violent and cruel. The absence of honor, to a great extent, may impede their perseverance and performance, making it challenging to demonstrate the martial spirit and heroic qualities. Honor is a kind of morality of aspiration, which contributes to the efficient functioning of the organization and the realization of its vision, thus fulfilling the martial virtue in the military context [42]. Related literature proposed that corporate social responsibility practices such as environmental investment can enhance entrepreneurs’ reputations, leading them to a sense of honor [43]. Their positive impression among stakeholders (including employees) also benefits from investment in corporate social responsibility practices such as environmental investment [44,45]. Therefore, when military entrepreneurs feel the external honor, they will be more motivated to prompt environmental protection of their enterprise.

The third is the willingness to contribute. The military emphasizes duty, dedication, and self-sacrifice. The entrepreneurs who work in the military have received rigorous training, good moral beliefs, and spirits of service and self-sacrifice [16,23]. Through the education of ideals and morality, the army can stimulate them to show a higher moral standard in corporate governance [16]. Accordingly, Wal-Mart and GE have recruited junior officers serving in Iraq and Afghanistan to solve the problem of lacking leadership and dedication of young managers [21]. Additionally, besides protecting and defending, the Chinese military also undertakes essential emergency public service functions such as medical support, voluntary blood donation, or flood relief, giving soldiers more opportunities to practice dedication, thereby enhancing the imprint of values. Therefore, driven by the military imprint, entrepreneurs are more likely to make contributions to environmental governance when they realize the serious environmental problems. Consequently, we propose Hypothesis 1:

**H1.**  *Ceteris paribus, firms with military entrepreneurs may make more environmental investments than their counterparts without military entrepreneurs.*

### 2.3. The Moderating Effect of Wartime Atmosphere

Imprinting theory provides a theoretical perspective for studying the relationship between military experience and environmental investment behavior. According to the viewpoint of imprinting theory, the imprint formed by individuals in the sensitive period has a firm consistency with the external environment [30,36]. The institutionalization process of imprinting depends not only on its imprinter, but also on external macro-environmental factors (e.g., social situation and atmosphere), which will affect the effect of the imprinter’s institutionalization process. Therefore, the wartime atmosphere, one of the macro-environment factors, may also affect that in the process of shaping values of new military members. For instance, Malmendier [20] found that military experienced Second World War had a more positive influence on management behavior.

The military experience in wartime has shaped the values of the soldiers more strongly than in the peace years because the war atmosphere highlights the social need for the sense of values of the soldiers. The imprint of military value education can be strengthened in three aspects: First, the army would enhance the education of military values to prepare for external threats. It will mobilize the soldiers more profoundly and extensively. Second, the war environment provides a real opportunity to practice values and guide individuals with some typical examples, thereby promoting the institutionalization of the imprint of values. In particular, front-line soldiers who accept heroism will have a deeper understanding of obedience, honor, and dedication, leading to a more profound ideological imprint. Third, the consistency of social wartime mobilization with the value education of the army will reduce interference, and hence it provides a good external environment for the value imprint of the military. Extensive propaganda and public opinion have further strengthened soldiers’ own role consciousness. Accordingly, we propose Hypothesis 2:

**H2.**  *Ceteris paribus, the effect of military entrepreneurs is more pronounced for firms with entrepreneurs who have experienced a wartime atmosphere.*

### 2.4. The Moderating Effect of Public Environmental Concern

Public environmental concern is one of the critical driving factors for environmental investment [46]. While the imprint formed by military experience also has a positive effect on environmental investment. The former is the pressure from the external benefits that entrepreneurs feel, and the latter is the internal cognitive traits shaped by their own extraordinary experiences. Both possess a positive effect of military imprint on environmental investment. However, rather than focusing on the direct effect of public environmental concern, we argue that public concern relative to the environment moderates the effect of entrepreneurs’ military experience on environmental protection under the influence of military imprint.

Specifically, when the public pays more attention to environmental protection, to increase consumers’ purchase intentions, companies will attempt to achieve non-altruistic objectives by increasing environmental investment [47]. For example, in areas where there are strong public demands in environmental improvement, entrepreneurs will invest more in environmental protection to avoid troubles and complaints, gain public goodwill and improve their corporate image so as to obtain better benefits. Under this circumstance, environmental investment is not a concern for social well-being, but a consideration of interests. We believe that military executives are reluctant to participate in these environmental investment activities because these are not derived from their inner pursuit of honor and dedication. Therefore, when entrepreneurs feel more significant external public pressure, the driving force brought by the imprint of military ideology for environmental investment will be weakened. In contrast, in areas where the public environmental concerns are relatively insufficient, the environmental investment may be more driven by altruistic motivation. In such cases, top executives are unlikely to engage in environmental investment to cater to public environmental concerns for gain benefits. However, driven by their military imprint, military executives may contribute to the solutions to the environmental issues out of their inner honor and dedication.

As such, the public environmental concern weakens the positive correlation between the military experience of entrepreneurs and corporate environmental investment. Instead, the positive effect of military imprint on environmental investment will be more significant when the public environmental concern is weak. As a result, we propose Hypothesis 3:

**H3.**  *Ceteris paribus, the effect of military entrepreneurs is more pronounced for firms located in areas of insufficient public environmental concern.*

## 3. Research Methods and Data

### 3.1. Data Source

We tested our hypotheses with the dataset from the tenth Chinese Private Enterprise Survey (CPES) of 2012. The survey is conducted biennially and organized by the United Front Work Department of the CPC Central Committee, the All-China Federation of Industry and Commerce (ACFIC), the State Administration for Industry and Commerce of the People’s Republic of China, and the Private Economy Research Institute of China. It began in 1992, the first year where the ruling party proposed the establishment of a socialist market economy. The survey conducted a multi-stage stratified sampling of private enterprises registered with the State Administration for Industry and Commerce (SAIC) at a rate of 0.55%. That was, selected counties and county-level cities according to the level of economic development, and then randomly select the entrepreneurs of the surveyed enterprises according to the distribution of urban and rural areas and industries. The final data were obtained from 5073 enterprises of different sectors and sizes from 31 provinces in China, widely used in many previous academic studies [48,49,50].

To ensure the reasonableness and validity of the data samples, strict screening was performed according to the following criteria: (1) eliminating samples with missing key variables. (2) Eliminating samples in the financial industry because they have different accounting standards. (3) Winsorizing the continuous variables at the top and bottom 1% of their distribution to mitigate the influence of potential outliers. The final sample consists of 2438 observations.

### 3.2. Model and Measurements

We established a benchmark regression model to explore the impact of entrepreneurs’ military experience on corporate environmental investment, as shown in the following Formula (1).

*Env*/*Denv* = β_0 + β_1*Sol* + β_2*Size* + β_3*Year* + β_4*Lev* + β_5*Profit* + β_6*Tax* + β_7*Edu* + β_8*Male* + β_9*Pay* + β_10*Own* + β_11*Age* + β_12*Inv* + β_13*Pol* + Industry + Region + ε (1)

When the dependent variable is *Denv*, we use the Probit model to conduct regression analysis because it is a binary virtual variable; in addition, considering that *Env* is censored dependent variable with left-censoring limit, which contains a considerable number of zero values, we thus use Tobit model to conduct regression analysis.

#### 3.2.1. Dependent Variable: Corporate Environmental Investment

The amount of corporate environmental investment defined in this paper is measured by the “How much did your company invest in pollution control in the past year?” in the Chinese Private Enterprise Survey questionnaire. This paper uses two methods: one is to use a dummy variable to measure (*Denv*), when the amount of corporate environmental investment is greater than 0, *Denv* is 1. Otherwise, it is 0; the other method is enterprise environmental investment plus 1, then take natural logarithm (*Env*).

#### 3.2.2. Independent Variable: Military Experience

The entrepreneur’s military experience (*Sol*) was measured using a direct code from the questionnaire: “What was your main experience before starting your business?”. If the military was selected, the code would be 1, otherwise, the code would be 0.

#### 3.2.3. Wartime Atmosphere

Since the founding of the People’s Republic of China in 1949, the Chinese Army has experienced the Korean War (1950–1953), the Vietnam War (1965–1973), the Sino-Indian border self-defense counterattack (1962), the Sino-Indian War (1969), and the Sino-Vietnamese War (1979). The measurement steps are as follows: First, determine the age of enlistment for men and women by the “Military Service Law of the People’s Republic of China”, in the meantime, considering soldiers who have served within two years before the start of the war; second, match the actual age of entrepreneurs and military experience with it according to the survey of the All-China Federation of Industry and Commerce. It should be pointed out that this calculation method can only determine whether an entrepreneur experiences a wartime atmosphere in the army, but cannot decide whether or not he participates in a war.

#### 3.2.4. Public Environmental Concern

Public environmental concern is measured by telephone/Internet complaints in each region. Telephones and the Internet are currently the main channels for the public to participate in environmental governance. The data is taken from the China Environment Yearbook.

#### 3.2.5. Control Variables

Following previous literature [17,21,51,52], the model control variables include two main categories: firm characteristics and individual entrepreneurial traits. Among them, enterprise characteristic variables include (1) enterprise size (*Size*), measured by the natural logarithm of the company’s annual operating income. (2) Established years (*Year*), measured as the natural logarithm of the number of years that have elapsed from the company’s registration to the year the survey took place. (3) Debt-to-asset ratio (*Lev*), measured as total liabilities divided by total assets. (4) Profitability (*Profit*), measured as net profit divided by operating income. (5) Tax payment (*Tax*), measured by dividing the sum of annual tax and various expenses by operating income. (6) Enterprise expansion (*Inv*), measured by the natural logarithm of the newly added investment plus 1 in the Chinese Private Enterprise Survey; entrepreneurs’ individual characteristics variables included. (7) Entrepreneur gender (*Male*), measured as the actual gender of the entrepreneur. If male, then it is 1. (8) Age (*Age*), measured as the actual age of the entrepreneur in 2012. (9) Education level (*Edu*), measured as the staged education completed by the entrepreneur. (10) Income (*Pay*), measured as total logarithm of entrepreneur’s income plus 1. (11) Shareholding ratio (*Own*), measured by the ratio of owner’s equity held by the entrepreneur. (12) Political connection (*Pol*), measured as 1 if the entrepreneur serves as a representative of the People’s Congress or a member of the CPPCC; otherwise, it is 0. At the same time, this article controls industry effects and regional effects.

## 4. Empirical Results

### 4.1. Summary Statistics

Table 1 reports descriptive statistics of the variables involved in the model. Table 1 reports the results of descriptive statistics for size, mean, median, standard deviation, minimum and maximum of the main variables. From the data in the table, we can see that the mean value of *Sol* is 0.430, which indicates that there are still a significant number of entrepreneurs who have military experience in private enterprises in China. The maximum value of *Env* is 5.994, the minimum value is 0, and the mean value is 0.855, while the maximum value of *Denv* is 1, the minimum value is 0, and the mean value is 0.350, which indicates that there is a significant difference in the amount of environmental investment in private enterprises in China, and some of them are not involved in any environmental input activities. *pol* is a binary variable with a mean value of 0.414 and a standard deviation of 0.493, which means that a large number of entrepreneurs in the sample have served as deputies to the National People’s Congress or members of the CPPCC in the government.

To further determine the differences between corporates run by military and nonmilitary entrepreneurs, we make a comprehensive comparison between these two types of corporates. As Table 1 Panel B shows, corporates led by military entrepreneurs significantly differ from their counterparts in corporate environmental investment. Specifically, on average, a corporate commits more resource to environment protection when its senior manager has military experience.

### 4.2. Correlation Analysis

Appendix B Table A1 and Table A2 reports the pairwise Pearson correlations among the main variables. As expected, military experience are positively related to independent variable, whether *Env* or *Denv*. Specifically, it is positively related to *Denv* at the 1% significance level, while *Env* at 5% significance level, which strengthens our hypotheses that military experience promotes corporate environmental investment. It also can be observed that corporate environmental investment has significant correlations with Enterprise size, Established years, Debt-to-asset ratio, Enterprise expansion, Entrepreneur gender, Age, Income, Shareholding ratio, Political connection, etc. These results suggest a need to control for these variables when examining the effect of military experience on corporate environmental investment. Additionally, the correlation among main variables is not higher than 0.50. Hence, multicollinearity is not a problem in our model. Additionally, to test the potential effect of multicollinearity, we calculate the variance inflation factor (VIF). Table 2 shows that most variables have a VIF less than 1.5, and the overall mean value is 1.34. This suggests that multicollinearity is not an issue in the model.

### 4.3. Regression Results

Table 3 presents the main results of the regression on the impact of military experience on corporate environmental investment. The dependent variable in models (1) and (2) is whether the firm has an environmental investment (*Denv*). The *Sol* regression coefficients are 0.347 before including the control variables in the model and 0.294 after, respectively significant at the 1% and 5% levels. Models (3) and (4) changed the dependent variable to (*Env*). When only the independent variable was included in the model, the *Env* regression coefficient was 0.323 with a significance level of 5%. After adding control variables to the model, the coefficient of *Env* is 0.279, and the significance level remains at 5%. These results show that the environmental investment of enterprises with military entrepreneurs is statistically higher than that of enterprises without military entrepreneurs, both before and after controlling entrepreneurs’ personal and corporate characteristics. These results support Hypothesis H1. In addition, the regression coefficients for part of the control variables are positive and highly significant in all models, indicating that larger firms, higher taxes, more new investment, and stronger political connection with the government are associated with the greater corporate environmental investment. Some of these results are supported by existing research findings [53], but the internal transmission paths of some results still require further design and empirical studies.

Table 4 reports empirical results of the moderating effects. We divide the sample into “experienced wartime atmosphere” group and “unexperienced wartime atmosphere” group according to whether they have experienced the wartime atmosphere, then put them into the regression equation, respectively. In the group of “experienced wartime” (Models 1 and 2), the regression coefficients of the dependent variables *Env* and *Denv* are 0.505 and 0.754, which are separately significant at the 5% and 1% levels. In contrast, in the group of “unexperienced wartime” (Models 3 and 4), the regression coefficients of the independent variables on *Env* and *Denv* are 0.199 and 0.143, but both of them are not significant. In addition, the regression coefficients of the independent variables are significantly larger in the “experienced wartime” group than in the “unexperienced wartime” group. These tests provided empirical support for the effect of wartime experience in Hypothesis H2.

Additionally, we divide the sample into the “high public environmental concern” group and the “low public environmental concern” group according to the median of public environmental concern, then put them into the regression equation, respectively. Table 4 shows that the regression coefficients of the dependent variables *Env* and *Denv* are 0.115 and −0.070 in regions with higher public environmental concerns (Models 1 and 2), which are both not significant. However, in areas with lower public environmental concerns (Models 3 and 4), the regression coefficients of the independent variables on *Env* and *Denv* are 0.633 and 0.432, which are both significant at the 1% level. In addition, the regression coefficients of the independent variables are significantly more prominent in the “low public environmental concern” group than in the “high public environmental concern” group. This indicates that when the public pays less attention to environmental issues, the imprint of entrepreneurs’ military experience will be strengthened, consistent with Hypothesis H3.

### 4.4. Robustness Checks

#### 4.4.1. Alternative Measures of Environmental Investment

We divide the natural logarithm of total investment in pollution control (*Env*) and the dummy variable of total investment in pollution control (*Denv*) by the natural logarithm of operating income and profit income to obtain *OIEnv*, *OIDenv*, *NPEnv*, and *NPDenv* as the original factors. We substitute new variables for original variables, plug the variables into the model for regression, and obtained the empirical results as shown in Table 5. It can be seen from Table 5 that when the dependent variables are *OIEnv*, *OIDenv*, and *NPEnv*, *NPDenv*, the regression coefficient of Sol before and after adding the control variables is always significant at the 5% level. The regression result is consistent with the main effect result, which proves that the entrepreneurial experience in the military has promoted the level of environmental investment of the enterprise. Therefore, the empirical conclusions of this article are still practical after replacing the measurement method of the dependent variable.

#### 4.4.2. Endogeneity Correction

Drawing on Rosenbaum and Rubin’s study (1983) [54], we use the PSM method to screen out the firms similar to those where the managers have military experience, but where the managers do not have military experience to examine the impact of military background executives on firms. This method matches entrepreneurs with military experience with those without military experience. There is no significant difference between the two types of firms after controlling for other factors, except for the presence or absence of military experience. Thus, reliable causal effects are inferred. The specific steps of propensity score matching (PSM) are as follows: firstly, a logistic model regression is conducted with all firm characteristics and individual entrepreneur characteristic variables except the independent variable, and the propensity score of whether entrepreneurs with military background manage the sample firms is estimated based on the logistic model; Then, according to the “Nearest neighbor matching” principle, the enterprises managed by entrepreneurs with military experience and enterprises without military experience were matched with control samples, and a total of 212 effective observation samples were obtained. Table 6 presents the correlation results after applying propensity score matching (PSM).

The results of the sample balance test before and after off-table matching showed that the validity of the matching was verified by eliminating the variability of all firm characteristic variables and individual entrepreneur characteristic variables after matching except for the independent variable. Panel A conducted a between-group difference test using PSM matched samples around the dependent variable environmental investment (*Env*, *Denv*). The results showed that the differences in firm characteristics between the experimental and control groups before matching were highly significant. At the same time, the sample differences were effectively controlled after PSM matching, and the sample was better balanced, which again verified hypothesis 1. Panel B reported the results of multiple regression analysis using matched samples. When the dependent variables were *Env* and *Denv*, the regression coefficients of Sol were 0.602 and 0.400, respectively, both at the 5% or higher level of significance, indicating that entrepreneurs’ experience in the military can significantly increase the level of corporate environmental investment, which remains consistent with the previous findings.

## 5. Discussion

### 5.1. Contributions

This study makes several contributions to the extant literature. First, we extend the literature on the determinants of corporate environmental investment. Existing studies have demonstrated concern of business efforts under the dual influence of profit-driven and environmental concerns [55]. However, concerning the characteristics of senior managers, especially in terms of their military experience, there is little evidence about its governance role in the firm’s environmental protection activities. Given that the important roles of imprint formed by the previous experience in entrepreneurs’ managerial behaviors [20,21], we investigate the effect of the imprint of military experience on corporate environmental investment to supplement the literature in the field of environmental governance and sharpens our understanding of the motivations of corporate environmental investment in emerging economies.

Furthermore, extant studies, based on behavioral and psychological literature, find that military experience can affect financial performance and investment decisions [20,21]. We extend this line of research by building on imprinting theory and upper echelon theory in that we demonstrate that service in military can serve as an indicator for obedience, honor, and dedication behavior, which matter in the context of corporate environmental responsibility. By proposing that entrepreneurs’ military service leaves a lasting imprint that leads to more corporate environmental investment, our study enriches the literature of how military background may impact corporate policies, especially corporate environmental decision-making.

Finally, this study mainly enriches the imprinting literature by highlighting the boundary condition between entrepreneurs’ previous military experience and corporate environmental investment. Imprinting theory has pointed out that external circumstances are crucial in understanding imprinting dynamics and the subsequent functions [30,39]. When imprints are generated or take effect, various functions from the wartime atmosphere and public environmental concern would be implemented. By taking advantage of them in this study, we find that the dynamic influence of external environment on individual imprint could strengthen or attenuate the positive effect of military entrepreneurs on corporate environmental investment, which provides a deeper understanding of imprint theory.

### 5.2. Practical Implications

Our research conclusions have several practical implications as well. First, our results offer a new approach to understanding why firms react so differently to environmental activities. More importantly, given the public’s increasing attention on corporate environmental responsibility, firms considering candidates for their top positions should conduct an in-depth investigation of candidates’ life experience, especially in a sensitive period. Second, our research responds to existing research [56] that believes that military experience is of great value outside the military environment and provided a new perspective on the importance to the country of the talent produced by the excellent management culture of the military. Moreover, this also provides practical insights for policymakers on how to implement environmental governance and promote ecological construction. Third, we provide a new perspective for solving the rational placement of military personnel after major disarmament and routine demobilized military personnel. Encouraging soldiers to start their businesses and giving preferential policies can solve the employment problem and improve social–environmental performance. Since military entrepreneurs have a unique imprint of the military, they will pay more attention to the country and society’s environmental issues. They are more likely to invest more in environmental governance while running their business.

## 6. Conclusions

In response to severe environmental degradation, countries are actively involved in environmental governance and will face enormous financial needs. As the main driving force of economic development, enterprises should undertake the due obligations of environmental protection. This study explores the impact of entrepreneurs’ military experience on corporate environmental investment and its boundaries in Chinese context. Based on an empirical investigation of the environmental investment of 2438 private firms, we establish an analysis model of the impact of military experience on corporate environmental investment referring to the existing literature, which provides an empirical basis for the research hypothesis proposed in this paper.

Based on the study, we draw the following three conclusions: (1) We hold the belief that military experience helps entrepreneurs form an imprint of values—discipline, honor, and dedication—which lead them to actively undertake social responsibilities. The empirical results found that entrepreneurs’ military experience has a significant positive impact on corporate environmental investment behavior; (2) a set of moderation tests reveal that wartime atmosphere, as an external macro-environmental factor, will affect the imprinter’s institutionalization process and further deepen the imprint of military experience on the values of military entrepreneurs. Thus, experience under a wartime atmosphere can reinforce the military imprint of military entrepreneurs and motivate them to increase their corporate environmental investments; (3) we find that the intensity of public environmental concern can effectively regulate the relationship between entrepreneurs’ military experience and corporate environmental investment. In regions with lower public environmental concerns, military experience has a more positive impact on corporate environmental investment. That probably is, entrepreneurs with military experience are more reluctant to use the corporate environmental investment for non-altruistic objectives.

More broadly, our study reveals that the entrepreneur imprint is an important driving force for corporate environmental behavior, which enriches the existing research on sustainable development and provides a more comprehensive understanding of corporate interests and environmental problems. Besides China, when other countries advance environmental governance, they can pay more attention to shaping entrepreneurs’ imprints in certain specific environments, encouraging environmental entrepreneurship and cultivating an excellent social atmosphere for corporate environmental behaviors.

We also need to pay attention to the limitations of this study. First, considering the availability of the current data, it is difficult to analyze the specific military characteristics, such as the types of service, ranks, and years of service, whose differences may bring more interesting theoretical and practical implications. More fine-grained data can be collected through questionnaires in the future. Besides, the military imprint discussed in this article is only one reason that drives corporates to make more environmental investments. Follow-up research could study other imprints that may substantially impact executives to obtain a more comprehensive understanding of corporate environmental investment by executives.

## Figures and Tables

**Table 1:** Summary statistics.

Panel A: Descriptive Statistics										
Variable	*N*		Mean		Median		S.D.		Min	Max
*Env*	2438		0.855		0		1.466		0	5.994
*Denv*	2438		0.350		0		0.477		0	1
*Sol*	2438		0.0430		0		0.204		0	1
*Size*	2438		6.855		7.010		2.365		1.194	11.66
*Year*	2438		9.252		9		5.275		1	22
*Lev*	2438		0.212		0		0.263		0	0.870
*Profit*	2438		0.0900		0.0460		0.168		−0.531	0.693
*Tax*	2438		0.0910		0.0530		0.136		0	0.900
*Edu*	2438		3.884		4		1.093		1	6
*Male*	2438		1.154		1		0.361		1	2
*Pay*	2438		2.528		2.398		0.923		0.693	5.303
*Own*	2438		0.768		0.962		0.293		0	1
*Age*	2438		46.10		46		8.697		25	69
*Inv*	2438		0.0300		0		0.0940		0	0.624
*Pol*	2438		0.414		0		0.493		0	1
**Panel B: Univariate Tests**										
	**Military Experience = 0**				**Military Experience = 1**				**Difference**	
	***N***	**Mean**		**S.D.**	***N***	**Mean**		**S.D.**		
*Denv*	106	0.472		0.502	2332	0.345		0.475	−0.127 ***	
*Env*	106	1.211		1.626	2332	0.838		1.457	−0.373 **	

Note: ***, ** and * indicate significance at the level of 1%, 5% and 10%, respectively.

**Table 2:** Variance inflation factors of main variables.

Variable	VIF	1/VIF
*Sol*	1.02	0.980210
*Size*	2.56	0.390030
*Year*	1.41	0.706716
*Lev*	1.29	0.776051
*Profit*	1.31	0.763006
*Tax*	1.32	0.756182
*Edu*	1.19	0.841570
*Male*	1.07	0.931029
*Pay*	1.44	0.694870
*Own*	1.07	0.934790
*Age*	1.25	0.798415
*Inv*	1.20	0.834949
*Pol*	1.34	0.747946

**Table 3:** The effect of military experience on corporate environmental investment.

Variables	(1)	(2)	(3)	(4)
	*Denv*	*Denv*	*Env*	*Env*
*Sol*	0.347 ***	0.294 **	0.323 **	0.279 **
	(2.61)	(2.13)	(2.36)	(2.23)
*Size*		0.169 ***		0.177 ***
		(8.07)		(10.34)
*Year*		0.022 ***		−0.001
		(3.48)		(−0.15)
*Lev*		−0.128		0.062
		(−1.04)		(0.57)
*Profit*		0.883 ***		0.082
		(3.94)		(0.47)
*Tax*		0.640 **		0.959 ***
		(2.52)		(4.48)
*Edu*		−0.042		−0.012
		(−1.47)		(−0.46)
*Male*		−0.056		−0.037
		(−0.63)		(−0.51)
*Pay*		0.031		0.079 **
		(0.85)		(2.40)
*Own*		−0.123		−0.200 **
		(−1.20)		(−2.25)
*Age*		−0.004		−0.004
		(−0.95)		(−1.32)
*Inv*		0.784 **		2.727 ***
		(2.38)		(9.30)
*Pol*		0.241 ***		0.173 ***
		(3.73)		(2.92)
*Constant*	0.018	−1.404 ***	0.841 ***	−0.381
	(0.18)	(−4.78)	(29.60)	(−1.64)
*Region*	Yes	Yes	Yes	Yes
*Industy*	Yes	Yes	Yes	Yes
*Observations*	2434	2434	2438	2438
*Pseudo R2 (R2)*	0.118	0.201	0.134	0.287

Note: ***, ** and * indicate significance at the level of 1%, 5% and 10%, respectively.

**Table 4:** The moderating effect of wartime atmosphere and public environmental concern.

Variables	Wartime Atmosphere				Public Environmental Concern			
	Experienced		Unexperienced		High		Low	
	(1)	(2)	(3)	(4)	(1)	(2)	(3)	(4)
	*Env*	*Denv*	*Env*	*Denv*	*Env*	*Denv*	*Env*	*Denv*
*Sol*	0.505 **	0.754 ***	0.199	0.143	0.115	−0.070	0.432 ***	0.633 ***
	(2.40)	(3.02)	(1.26)	(0.80)	(0.60)	(−0.34)	(2.62)	(3.31)
*Controls*	0.136 ***	0.107 **	0.187 ***	0.200 ***	0.214 ***	0.196 ***	0.136 ***	0.123 ***
	(3.71)	(2.48)	(9.57)	(8.11)	(7.93)	(6.20)	(5.95)	(4.20)
*Year*	0.004	0.019	−0.001	0.027 ***	0.001	0.025 ***	−0.004	0.018 *
	(0.34)	(1.52)	(−0.13)	(3.52)	(0.14)	(2.87)	(−0.51)	(1.85)
*Lev*	0.012	0.003	0.094	−0.145	0.094	−0.083	0.058	−0.209
	(0.05)	(0.01)	(0.73)	(−1.00)	(0.60)	(−0.49)	(0.37)	(−1.10)
*Profit*	−0.078	0.083	0.122	1.168 ***	0.279	0.828 **	0.010	1.029 ***
	(−0.20)	(0.18)	(0.63)	(4.44)	(0.95)	(2.37)	(0.05)	(3.37)
*Tax*	0.991 **	1.034 *	0.943 ***	0.552 *	1.097 ***	0.616 *	0.585 *	0.538
	(2.05)	(1.88)	(3.91)	(1.90)	(3.61)	(1.81)	(1.88)	(1.31)
*Edu*	0.072	0.079	−0.036	−0.084 **	−0.018	−0.012	0.003	−0.060
	(1.38)	(1.35)	(−1.22)	(−2.52)	(−0.49)	(−0.30)	(0.09)	(−1.41)
*Male*	0.081	−0.107	−0.074	−0.050	0.050	0.014	−0.086	−0.110
	(0.48)	(−0.50)	(−0.92)	(−0.52)	(0.45)	(0.11)	(−0.89)	(−0.87)
*Pay*	0.127 *	0.126 *	0.065 *	−0.004	0.111 **	0.037	0.022	0.013
	(1.95)	(1.75)	(1.70)	(−0.08)	(2.39)	(0.74)	(0.46)	(0.22)
*Own*	−0.330 *	0.057	−0.159	−0.185	−0.243 *	−0.115	−0.195	−0.215
	(−1.75)	(0.26)	(−1.56)	(−1.57)	(−1.79)	(−0.77)	(−1.63)	(−1.45)
*Age*	0.011	0.012	−0.004	−0.010 *	−0.002	−0.002	−0.006	−0.004
	(0.96)	(0.95)	(−0.85)	(−1.65)	(−0.43)	(−0.48)	(−1.25)	(−0.73)
*Inv*	2.848 ***	0.553	2.625 ***	0.727 *	3.198 ***	0.741	2.339 ***	1.023 **
	(4.68)	(0.79)	(7.77)	(1.93)	(7.35)	(1.52)	(5.87)	(2.19)
*Pol*	0.175	0.353 ***	0.193 ***	0.230 ***	0.039	0.135	0.348 ***	0.403 ***
	(1.51)	(2.73)	(2.74)	(2.98)	(0.48)	(1.57)	(3.94)	(3.96)
*Constant*	−1.454 *	−2.591 ***	−0.323	−1.141 ***	−0.873 **	−1.722 ***	0.057	−1.100 ***
	(−1.95)	(−2.93)	(−1.13)	(−3.14)	(−2.41)	(−3.92)	(0.18)	(−2.64)
*Region*	Yes	Yes	Yes	Yes	Yes	Yes	Yes	Yes
*Industry*	Yes	Yes	Yes	Yes	Yes	Yes	Yes	Yes
*Observations*	588	574	1849	1846	1259	1255	1178	1175
*Pseudo R2 (R2)*	0.281	0.1853	0.298	0.2188	0.294	0.1915	0.293	0.2295

Note: ***, ** and * indicate significance at the level of 1%, 5% and 10%, respectively.

**Table 5:** Robustness checks of replacing the dependent variable.

Variables	(1)	(2)	(3)	(4)
	*OIEnv*	*OIDenv*	*NPEnv*	*NPDEnv*
*Sol*	0.036 **	0.294 **	0.061 **	0.293 **
	(2.38)	(2.13)	(2.10)	(1.98)
*Size*	0.014 ***	0.169 ***	0.021 ***	0.174 ***
	(6.99)	(8.07)	(5.09)	(7.53)
*Year*	0.000	0.022 ***	0.000	0.019 ***
	(0.12)	(3.48)	(0.15)	(2.86)
*Lev*	0.007	−0.128	0.033	−0.198
	(0.51)	(−1.04)	(1.30)	(−1.52)
*Profit*	0.040 *	0.883 ***	−0.105 **	0.990 ***
	(1.92)	(3.94)	(−2.17)	(3.67)
*Tax*	0.149 ***	0.640 **	0.225 ***	0.663 **
	(5.74)	(2.52)	(4.42)	(2.50)
*Edu*	−0.001	−0.042	−0.003	−0.044
	(−0.41)	(−1.47)	(−0.61)	(−1.47)
*Male*	−0.006	−0.056	−0.009	−0.052
	(−0.74)	(−0.63)	(−0.56)	(−0.56)
*Pay*	0.010 ***	0.031	0.002	0.029
	(2.60)	(0.85)	(0.24)	(0.76)
*Own*	−0.021 **	−0.123	−0.051 **	−0.156
	(−1.98)	(−1.20)	(−2.48)	(−1.46)
*Age*	−0.000	−0.004	−0.001	−0.005
	(−1.12)	(−0.95)	(−1.58)	(−1.20)
*Inv*	0.230 ***	0.784 **	0.261 ***	0.689 **
	(6.48)	(2.38)	(3.99)	(2.04)
*Pol*	0.022 ***	0.241 ***	0.042 ***	0.259 ***
	(3.02)	(3.73)	(3.09)	(3.86)
*Constant*	−0.008	−1.404 ***	0.100 *	−1.318 ***
	(−0.29)	(−4.78)	(1.84)	(−4.25)
*Region*	Yes	Yes	Yes	Yes
*Industry*	Yes	Yes	Yes	Yes
*Observations*	2438	2434	2211	2207
*Pseudo R2 (R2)*	0.234	0.201	0.183	0.195

Note: ***, ** and * indicate significance at the level of 1%, 5% and 10%, respectively.

**Table 6:** Robustness checks of propensity score matching.

Panel A: Balance Test						
PSM	Variable	Treated	Control	(%) Bias	*t*	*p* > |t|
unmatched	*Size*	7.1407	6.8739	11.7	1.14	0.256
	*Year*	10.321	9.2196	20.2	2.10	0.036
	*Lev*	0.21031	0.21441	−1.6	−0.16	0.876
	*Profit*	0.10098	0.08985	5.7	0.67	0.504
	*Tax*	0.09569	0.09039	3.9	0.40	0.692
	*Edu*	3.8396	3.8718	−3.0	−0.30	0.767
	*Male*	1.0566	1.1542	−32.1	−2.75	0.006
	*Pay*	2.5979	2.5282	7.3	0.76	0.448
	*Own*	0.7052	0.7697	−20.6	−2.21	0.027
	*Age*	47.83	46.062	19.8	2.05	0.041
	*Inv*	0.0209	0.03055	−11.9	−1.02	0.307
	*Pol*	0.5	0.4137	17.3	1.76	0.078
matched	*Size*	7.1407	6.9786	7.1	0.51	0.611
	*Year*	10.321	10.443	−2.3	−0.17	0.868
	*Lev*	0.21031	0.2024	3.0	0.23	0.820
	*Profit*	0.10098	0.09693	2.1	0.15	0.882
	*Tax*	0.09569	0.10074	−3.7	−0.24	0.808
	*Edu*	3.8396	3.8208	1.7	0.13	0.898
	*Male*	1.0566	1.066	−3.1	−0.28	0.776
	*Pay*	2.5979	2.5209	8.0	0.56	0.576
	*Own*	0.70524	48.67	−9.4	−0.35	0.723
	*Age*	47.83	0.02176	−1.1	−0.68	0.497
	*Inv*	0.0209	0.0218	−11.9	−0.09	0.927
	*Pol*	0.5	0.5	0.0	0.00	l.000
**Panel B: Differences Test between Groups**						
**Variable**	**Treated**	**N**	**Control**	**N**	**Difference**	***t*-Test**
*Env*	1.211	106	0.787	106	0.424 ***	2.44
*Denv*	0.472	106	0.302	106	0.066 **	2.04
**Panel C: Regression Results of PSM**						
**Variable**	***Env***			***Denv***		
*Sol*	0.602 *** (2.62)			0.400 ** (2.2)		
*Size*	0.281 *** (3.3)			0.249 *** (3.99)		
*Year*	0.015 (0.65)			−0.018 (−0.89)		
*Lev*	−0.296 (−0.56)			−0.302 (−0.73)		
*Profit*	0.723 (0.91)			−0.807 (−1.33)		
*Tax*	1.39 (1.26)			1.776 ** (2.3)		
*Edu*	−0.015 (−0.13)			−0.035 (−0.38)		
*Male*	−1.142 * (−1.66)			−0.305 (−0.77)		
*Pay*	0.082 (0.58)			0.069 (0.6)		
*Own*	−0.163 (−0.42)			−0.397 (−1.30)		
*Age*	−0.011 (−0.71)			−0.003 (−0.28)		
*Inv*	1.175 (0.63)			1.935 (1.33)		
*Pol*	0.141 (0.55)			−0.009 (−0.04)		
*_cons*	−1.311 (−0.92)			−0.109 (−0.12)		
*Industry*	Yes			Yes		
*Region*	Yes			Yes		
*N*	212			212		
*Adj.R_2_*	0.331			0.287		

Note: ***, ** and * indicate significance at the level of 1%, 5% and 10%, respectively.

## Data Availability

The data presented in this study are available on request from the corresponding author. The data are not publicly available due to data publisher regulations.

## Appendix A

Footnote 1. Notwithstanding Washington has proclaimed its withdrawal, the US chose to rejoin the agreement in 2021.

Footnote 2. Based on the latest data from the National Bureau of Statistics of China.

Footnote 3. According to the Annual Report on Ecological and Environmental Statistics (2020), industrial emissions of sulfur dioxide, nitrogen oxides, and dust from enterprises account for 86.5%, 44.4%, and 85.1% of total social emissions.

Footnote 4. For example, Ren Zhengfei, the president of Huawei, built a communications empire; Liu Chuanzhi, the founder of Lenovo Group, set up the world’s biggest computer companies; Zheng Yonggang, the chairman of Shanshan, created a famous brand of Chinese clothing; and Ning Gaining, the chairman of China Sinochem Corporation, is known as China’s J.P. Morgan.

## Appendix B

**Table A1:** Pearson correlation matrix of the dependent variable *Denv*.

	Denv	Sol	Size	Year	Lev	Profit	Tax	Edu	Male	Pay	Own	Age	Inv	Pol
Denv	1													
Sol	0.054 ***	1												
Size	0.330 ***	0.0260	1											
Year	0.203 ***	0.043 **	0.407 ***	1										
Lev	0.125 ***	−0.00100	0.405 ***	0.140 ***	1									
Profit	0.067 ***	0.0140	−0.164 ***	−0.0100	−0.197 ***	1								
Tax	0.00300	0.00700	−0.299 ***	−0.060 ***	−0.134 ***	0.379 ***	1							
Edu	0.0290	−0.00900	0.220 ***	0.044 **	0.085 ***	−0.0230	−0.0230	1						
Male	−0.096 ***	−0.057 ***	−0.172 ***	−0.113 ***	−0.089 ***	−0.0130	0.0180	0	1					
Pay	0.192 ***	0.0160	0.432 ***	0.255 ***	0.059 ***	0.186 ***	−0.00100	0.158 ***	−0.109 ***	1				
Own	−0.054 ***	−0.045 **	−0.108 ***	0.047 **	−0.110 ***	0.060 ***	0.00300	−0.116 ***	−0.00800	0.051 **	1			
Age	0.104 ***	0.042 **	0.236 ***	0.358 ***	0.138 ***	−0.063 ***	−0.036 *	−0.160 ***	−0.069 ***	0.096 ***	−0.0100	1		
Inv	0.200 ***	−0.0200	0.368 ***	0.138 ***	0.193 ***	−0.0150	−0.0270	0.159 ***	−0.080 ***	0.217 ***	−0.056 ***	0.072 ***	1	
Pol	0.245 ***	0.037 *	0.436 ***	0.300 ***	0.141 ***	0.00700	−0.055 ***	0.134 ***	−0.109 ***	0.275 ***	0.0270	0.158 ***	0.224 ***	1

Note: ***, ** and * indicate significance at the level of 1%, 5% and 10%, respectively.

**Table A2:** Pearson correlation matrix of the dependent variable *Env*.

	Env	Sol	Size	Year	Lev	Profit	Tax	Edu	Male	Pay	Own	Age	Inv	Pol
Env	1													
Sol	0.052 **	1												
Size	0.418 ***	0.0260	1											
Year	0.171 ***	0.043 **	0.407 ***	1										
Lev	0.194 ***	−0.00100	0.405 ***	0.140 ***	1									
Profit	0.00700	0.0140	−0.164 ***	−0.0100	−0.197 ***	1								
Tax	−0.00700	0.00700	−0.299 ***	−0.060 ***	−0.134 ***	0.379 ***	1							
Edu	0.083 ***	−0.00900	0.220 ***	0.044 **	0.085 ***	−0.0230	−0.0230	1						
Male	−0.105 ***	−0.057 ***	−0.172 ***	−0.113 ***	−0.089 ***	−0.0130	0.0180	0	1					
Pay	0.237 ***	0.0160	0.432 ***	0.255 ***	0.059 ***	0.186 ***	−0.00100	0.158 ***	−0.109 ***	1				
Own	−0.089 ***	−0.045 **	−0.108 ***	0.047 **	−0.110 ***	0.060 ***	0.00300	−0.116 ***	−0.00800	0.051 **	1			
Age	0.095 ***	0.042 **	0.236 ***	0.358 ***	0.138 ***	−0.063 ***	−0.036 *	−0.160 ***	−0.069 ***	0.096 ***	−0.0100	1		
Inv	0.326 ***	−0.0200	0.368 ***	0.138 ***	0.193 ***	−0.0150	−0.0270	0.159 ***	−0.080 ***	0.217 ***	−0.056 ***	0.072 ***	1	
Pol	0.255 ***	0.037 *	0.436 ***	0.300 ***	0.141 ***	0.00700	−0.055 ***	0.134 ***	−0.109 ***	0.275 ***	0.0270	0.158 ***	0.224 ***	1

Note: ***, ** and * indicate significance at the level of 1%, 5% and 10%, respectively.
